# Allogeneic Islet Transplantation: Chronicle of a Death Foretold?

**DOI:** 10.3389/ti.2025.14598

**Published:** 2025-04-01

**Authors:** Thierry Berney, Olivier Thaunat, Ekaterine Berishvili

**Affiliations:** ^1^ Department of Transplantation, Nephrology and Clinical Immunology, Edouard Herriot Hospital, Hospices Civils de Lyon, Lyon, France; ^2^ Faculty Diabetes Center, University of Geneva Medical Center, University of Geneva, Geneva, Switzerland; ^3^ Institute of Medical and Public Health Research, Ilia State University, Tbilisi, Georgia; ^4^ Centre International de Recherche en Infectiologie, INSERM U1111, Université Claude Bernard Lyon I, CNRS UMR5308, Ecole Normale Supérieure de Lyon, University Lyon, Lyon, France; ^5^ Lyon-Est Faculty of Medicine, Claude Bernard University (Lyon 1), Villeurbanne, France; ^6^ Department of Surgery, Laboratory of Tissue Engineering and Organ Regeneration, University of Geneva, Geneva, Switzerland; ^7^ Department of Surgery, Cell Isolation and Transplantation Center, Geneva University Hospitals and University of Geneva, Geneva, Switzerland

**Keywords:** islet transplantation, bioengineering, stem cells, xenotransplantation, artificial insulin delivery systems

## Abstract

Innovative solutions have entered the routine management of patients with type 1 diabetes or are making the headlines and this is shaking the world of beta cell replacement therapies. Above all, allogeneic islet transplantation is enthusiastically doomed to extinction by the aficionados of “closed loop” artificial insulin delivery systems or those convinced of the imminent large scale availability of stem-cell derived insulin-producing tissues. This opinion paper will propose that neither will be a universal solution in the very near future and will argue that xenogeneic islet transplantation may be a serious outsider in the race for new therapies. In the meantime, the odds are in favor of allogeneic islet (and pancreas) transplantation remaining first line options in the treatment of complicated type 1 diabetes. There is no question that “closed loop” systems have already greatly improved the management of type 1 diabetes, but, while “unlimited” sources of insulin-producing cells are jockeying for approval as standard-of-care, these improvements are more likely to drive a shift of indications -from islet transplant alone to simultaneous islet-kidney transplantation- than to herald the demise of islet transplantation.

## Introduction

Groundbreaking advancements are transforming the standard care of patients with type 1 diabetes mellitus (T1DM), sending ripples through the field of beta cell replacement therapies. Allogeneic islet transplantation, once hailed as a breakthrough, now faces existential questions amid the rise of stem cell-derived insulin-producing tissues and advanced closed-loop systems. There is a trend to believe that “closed-loop” artificial insulin delivery systems or stem cell-derived insulin-producing tissues will soon become the standard-of-care, thus limiting the remaining lifespan of islet transplantation. This opinion paper contends that allogeneic islet transplantation will persist as a key therapeutic option in the foreseeable future, not merely as a stopgap but as a complementary strategy within a diversifying armamentarium. When its decline eventually comes, if at all, the driving force behind it may not be one of the usual suspects.

## The Challengers (1)

**FIGURE 1:**
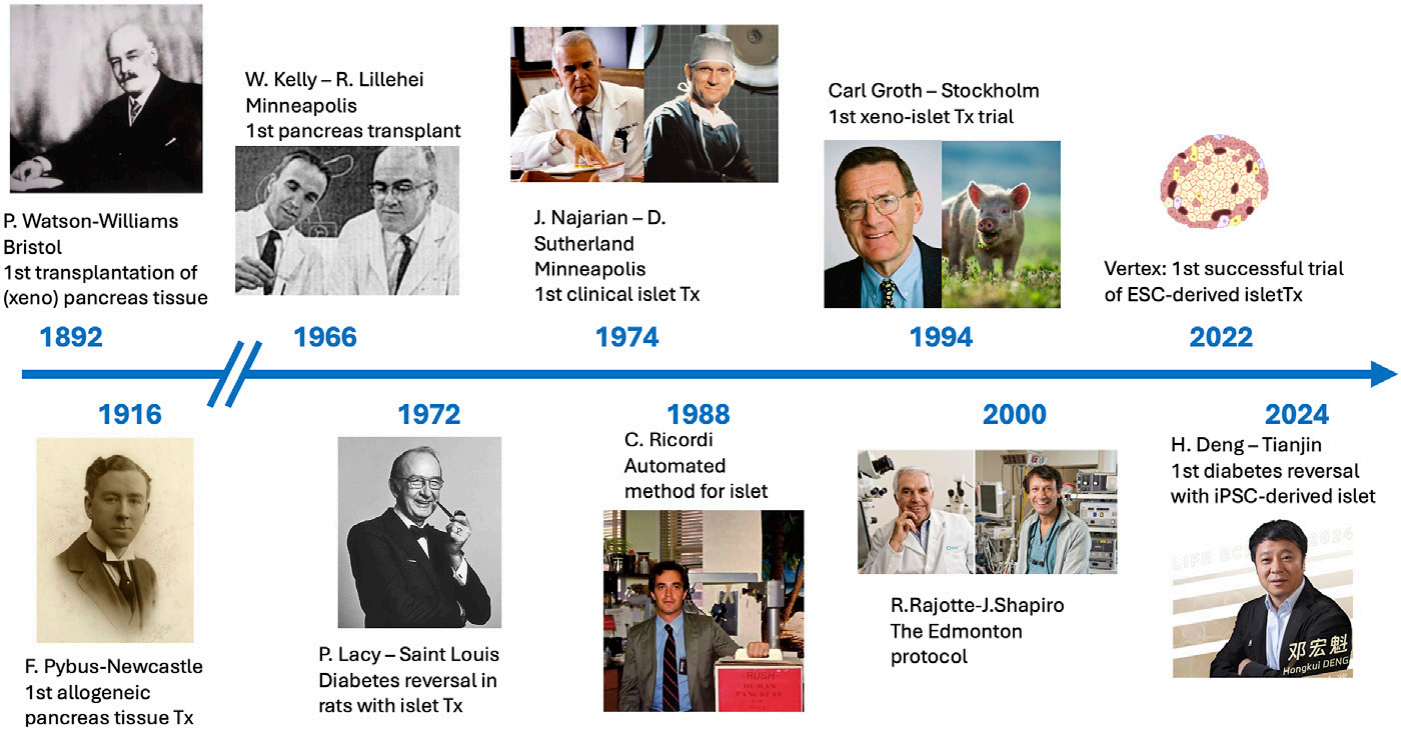
Timelines of beta-cell replacement therapies, from the first attempt at implanting sheep pancreas fragments into a boy with keto-acidosis in 1892 by Patrick Watson-Williams to the successful transplant of iPSC-derived islets by Honghui Deng, reported in 2024.

A revolution is in the making in the world of beta-cell replacement (Figure 1). The past 2 decades have seen sustained progress in the generation of insulin producing islet-like structures, derived from embryonic (ESC) or induced pluripotent (iPSC) stem-cells, exhibiting a fully mature β-cell phenotype and able to reverse diabetes in a variety of animal models [1–7].

The first phase I/II clinical trials of ESC-derived islet cells encapsulated in a macrodevice, developed by the Viacyte company, and transplanted to T1DM patients with or without immunosuppression depending on the device structure, essentially demonstrated tolerability and safety, notably absence of off-target growth or occurrence of teratoma [8, 9]. However, only minimal amounts of C-peptide were detected in less than half the study subjects, even after optimization of the number of transplanted cells [10]. The double hurdle of assessing at the same time cells still at the progenitor stage and an immune-isolating device may have accounted for these less-than-ideal results.

Meanwhile, the Vertex company designed 2 clinical trials, in which ESC-derived islet-like cell, developed from the works of the Harvard Stem Cell Institute [3], were transplanted to patients with T1DM. Importantly, these VX-880 cells are fully mature. The first Vertex trial, in which VX-880 cells were transplanted into the portal vein -as in clinical islet transplantation- and with immunosuppression, have demonstrated impressive results. In their latest press release, Vertex announced that islet cell engraftment and glucose-responsive insulin production occurred in all subjects. Nearly all participants (11 of 12) had a reduction or elimination of exogenous insulin use at their last visit, and all three patients who had reached at least 1 year of follow-up had come off insulin [11]. These remarkable results have allowed Vertex to announce the approval to move this trial to phase III [12]. A second trial in which the same cells are transplanted inside macrodevices without immunosuppression has been launched in the meantime.

Similarly spectacular clinical observations, albeit on a smaller scale, were reported from China, using iPSC-derived islet cells as the source of insulin-producing tissue. Chemically induced iPSC-derived autologous islets [7] were transplanted in a patient with T1DM, who was already on immunosuppression for a previous liver transplant. At 1-year post-transplant, patient was off-insulin, with normal blood sugar levels (time in range 99%) and normal HbA1c [13]. It is difficult to predict whether the autologous transplanted cells would have been protected from immune rejection or prone to recurrence of autoimmunity without immunosuppression.

Another group in China, reported the outcomes of a type 2 diabetic patient, already transplanted with a kidney and therefore on immunosuppression, in whom iPSC-derived islets were transplanted intraportally. Again, with more than 2 years follow-up, the patient remained off insulin, with normal blood sugar levels (time in range 99%) and normalized HbA1c [14]. Although its breakthrough nature was acknowledged, this report was met with cautious optimism, notably regarding the immunogenic profile of autologous iPSC-derived cells and their fate in the absence of immunosuppression [15]. Indeed, in contrast with ESCs that grow into teratomas into mice of the same genetic background, autologous iPSCs, reprogrammed from fetal fibroblasts by viral or non-viral genetic approaches, elicit an unexpected immune reaction in genetically identical mice, resulting in their rejection [16].

In an opinion paper published in the same issue of Transplant international, L. Piemonti discusses why, in spite of these spectacular breakthroughs, the large-scale application of stem cell therapy as a “cure” for T1DM may still face considerable hurdles before coming into implementation [17]. Large scale application, i.e. to “all” patients with T1DM before they develop complications of the disease in the form of severe hypoglycemia or micro/macrovascular disease, will require circumventing the need for lifelong immunosuppression. Solutions may include immune-isolating encapsulation systems and localized immunomodulation of the graft microenvironment or of the implanted cells themselves, rendering them “invisible” to the immune system by gene editing technologies [18–22]. However, translating these strategies into clinically viable Advanced Medicinal Therapy Products (ATMP), as they are classified in the European regulation, will demand significant technical and regulatory efforts, entail important costs, require cross-sector collaboration among all stakeholders -including academia, industry, healthcare systems, physicians, patient advocacy groups - and will take considerable time [23, 24].

A critical gap remains the lack of a “quality by design” approach, wherein diabetes-curing ATMPs are conceptualized holistically from inception—integrating cellular components, delivery systems, and immune protection—rather than retrofitting specific innovations into existing platforms *post hoc* [17]. For instance, the Vertex’s VX-880 product, a leading ESC-derived islet therapy, has shown remarkable early efficacy in Phase I/II trial, but its reliance on immunosuppression and its high production costs will likely restrict access to a privileged minority in the foreseeable future.

## The Challengers (2)

**FIGURE 2:**
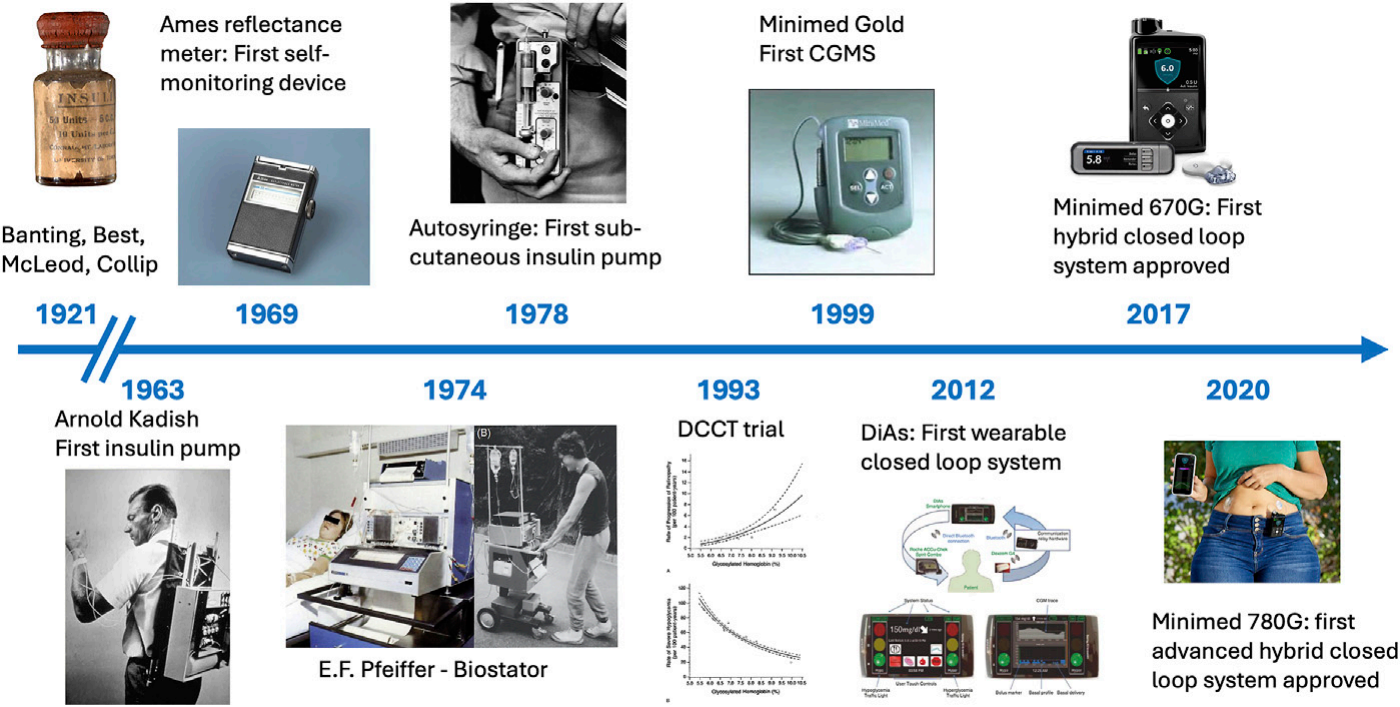
Timelines of insulin therapy, from its “discovery” in 1921 by Frederick Banting and Charles Best to the approval of the first advanced hybrid closed loop system in 2020.

The quest for a fully functional, fully autonomous “artificial pancreas” has relied on the parallel development, since the 1960s, of glucose sensors, which have evolved into continuous glucose monitoring (CGM) systems and of insulin delivering pumps [25] (Figure 2). The combination of these two technologies into what are known as “hybrid closed loop systems” is now part of the standard of care of patients with T1DM in industrialized countries. These systems rely on the measure (sensing) of subcutaneous glucose levels, which are entered into an algorithm that in turn determines the dose of insulin to inject subcutaneously. The “hybrid” terminology relates to the fact that, although the loops can effectively be closed, they still require input from the patient about carbohydrate intake or physical activity to complement the automated component of the system. The more recent generation, termed “advanced hybrid closed loop” systems (AHCL) have been approved by healthcare systems since 2020.

AHCL systems are extremely effective at improving glycemic control. Several studies with “real world” patients (i.e., not subject to the strict inclusion/exclusion criteria of randomized trials) have demonstrated a significant improvement of the glycemic time in range (TIR; 70–180 mg/dL), reaching 72%–74%, and HbA1c of approximately 7%, with 1-year follow-up periods [26–28].

AHCL systems have markedly improved both disease management and glycemic control of patients with T1DM. However admirable these achievements are, they should not conceal that the TIR targeted by diabetologists is not equate the normal glycemic range they have defined themselves. Investigators having looked at the time in “tight” range (70–140 mg/dL) obtained by AHCL systems, showed that it was in fact only 43%, even though a TIR of 73% was achieved [29]. The 7% HbA1c obtained, which is in line with accepted diabetologic targets, is in fact not better than the results of the DCCT/EDIC trials, which showed that intensive insulin therapy resulting in mean HbA1c of∼ 7% maintained over a mean 6.5 years reduced the development and progression of early microvascular complications associated with diabetes by 34%–76% [30]. New diabetes treatment technologies have thus resulted in a progressive slowing down of the development of end-stage nephropathy in patients with T1DM; as reported in a Swedish cohort, the onset of end-stage renal failure has been postponed at least 10 years compared with that in older prospective cohort studies [31].

From the patient perspective, AHCLs are generally very favorably considered, although a recent study reported that it did not improve diabetes treatment satisfaction, diabetes-specific quality of life, hypoglycemia awareness, or perceived frequency of unacceptably low glucose levels in study subjects [32]. Acceptability of AHCL is not universal (sensor issues, sports, …) and in some cohorts, the percentage of dropout from AHCLs was up to 30% [33].

In other words, and as already expressed by F. Banting in his Nobel acceptance speech, “insulin is not a cure, it is a treatment” [34]. No matter how sophisticated the AHCL device and the algorithm governing it are, the beta cell, and all the crosstalk and interactions that occur between the various cellular components of an islet of Langerhans, cannot be mimicked by a glucose sensor connected to insulin pump [35].

## The Outsider

The field of xenotransplantation has recently garnered significant attention due to the breakthrough transplantation of porcine kidneys and hearts into brain-dead human subjects (the decedent model) and living patients [36–40]. Encouraging, and even spectacular, results have been largely achieved thanks to the availability of genome-edited pigs, with genetic modifications knocking-out genes related to carbohydrate antigens known to cause hyperacute rejection and human transgene insertions, designed to modulate the human immune system [41, 42].

It is quite strange to observe that islets have not yet joined this bandwagon, since it has long been considered the ideal modality for a potential first successful xenotransplantation trial [43]. The technical aspects of an islet transplant much easier than those of a vascularized organ transplant and the consequences of a failed graft are much less dramatic. Additionally, porcine insulin differs from human insulin by only one amino acid and has been the mainstay of T1DM management for decades before the arrival of synthetic insulins. Unsurprisingly, early trials using wild-type or minimally modified porcine islets, often with suboptimal encapsulation strategies, unsurprisingly yielded poor outcomes [44].

Another interesting feature of islet grafts is that they are disconnected from their own vascularization at the time of implantation, and revascularized with vessels growing from the host over the first weeks of engraftment [45, 46]. This means that there is no encounter of the donor epithelium with the host antibodies, and therefore some extent of protection from antibody-mediated rejection [46]. These experimental observations have indeed been largely verified in the clinical field, in which no correlation was seen between occurrence of *de novo* donor-specific antibodies and islet graft loss [47, 48]. Thus, the humoral component of xenorejection, which is thought to be the major immunological hurdle for graft survival is likely to be of no consequence in islet xenotransplantation.

How close are we to efficacious clinical islet xenotransplantation [49]? In recent years, several groups have reported long-term islet graft survival in pig-to-nonhuman primate experiments, mostly using wild type adult pigs as donors [44]. The government of South Korea has invested significant funding to advance the field of islet xenotransplantation, and a sponsor-initiated trial (Seoul National University), using islets from pathogen-free, wild type adult pigs was approved the authorities and should be initiated shortly [44]. The pilot study will enroll 2 patients, with an immunosuppression protocol associating induction with T-cell and B-cell depletion and TNF and IL-1 blockade, and maintenance with tacrolimus and sirolimus, the former to be switched to JAK inhibitors at about 2 months [50].

Meanwhile, the Sydney group has recently achieved long-term porcine islet graft survival, well over 1 year, in nonhuman primates, using multigene-edited pigs and less heavy immunosuppression [51]. It seems that bringing islet transplantation to the clinic with acceptable immunosuppressive regimens will depend on the availability of genetically modified pigs and a better definition of which are the genes necessary (and sufficient) to edit in or out [42, 49, 52, 53].

Bringing islet xenotransplantation to the clinic will also require the resolution of regulatory issues, notably pertaining to biosafety in general, and specifically zoonosis transmission [54]. Despite initial concerns in the pioneering times, transmission of porcine endogenous retroviruses (PERV) has in fact never been observed, is easy to monitor and can be totally prevented by the now available pig in which the 57 PERV genes have been edited out [55]. Of greater concern is the risk of porcine CMV (in fact a porcine roseolovirus, PCMV/PRV) transmission, for which no treatment is known, and which has drastically reduced survival in pig-to-non human primates [41, 56, 57]. Although it is easy to breed pigs in PCMV/PRV-free conditions and this virus can be easily detected (PCR, serologies) [57], PCMV/PRV is likely to have been involved in the death of the first recipient of a porcine heart [58].

Islet xenotransplantation stands at a crossroads. Its unique biological advantages, coupled with advancing genetic and immunosuppressive tools, position it as a promising “outsider” in the race for scalable diabetes therapies. While technical and regulatory hurdles persist, the convergence of bioengineering innovation and clinical experience may yet propel islet xenotransplantation from theoretical promise to practical reality.

## Allogeneic Islet Transplantation: Quo Vadis?

The authors of this point of view hope to have convinced the reader that despite the recent reported successes, stem cell-derived islets are unlikely to become available to a large patient population in a so near future. Although, the proof of concept was spectacularly obtained in the recent Vertex trial, incorporation of the cells into a finalized immune-protected system still has to be achieved. It should also be mentioned that, although off-target cell proliferation has not been observed so far, it remains a potential hazard that, if verified, would set the field many years back.

If investigators engaged in the field of islet xenotransplantation are careful to engage early enough in the “quality by design approach” advocated by Piemonti [17], islet xenotransplantation might find itself having an edge in the pursuit for an “infinite” source of insulin-producing tissue, available to all patients with T1DM without the need for lifelong immunosuppression.

In this context, the “quality by design approach” refers to a bioengineering strategy that holistically addresses the key challenges of functionality, safety, biocompatibility, immune-protection, ease of implantation and retrieval, cost efficiency, and patient acceptability [59]. These factors are essential prerequisites for designing and constructing a bioartificial pancreas, regardless of whether the insulin-producing tissue is derived from stem cells or xenogeneic sources [45, 60–63].

Meanwhile, we hope to have shown that the closed-loop systems, rhetorically referred to as an “artificial pancreas,” are in fact simply a way -albeit a sophisticated one-of administering exogenous insulin, and are no more a cure for type 1 diabetes than dialysis is a cure for kidney failure. AHCLs can minimize the risk of severe hypoglycemia, and help keeping sugar levels “in range” about 70% of the time, allowing patients to maintain HbA1c levels at around 7%. This is more than bettered by islet transplantation, which keeps patients in a truly physiologic range for a higher part of the time [64], and for which follow-up data as long as 20 years are now available [65].

What then are the perspectives for allogeneic islet transplantation as a clinical activity, in the years to come, arguably for longer than predicted by some? Allogeneic islet transplantation has of course its limitations, primarily the scarcity of donors and the need for lifelong immunosuppression, carrying infectious, tumoral and nephrotoxicity risks.

Since the publication of the seminal “Edmonton protocol” paper, islet-transplant-alone (ITA) for severe hypoglycemia/hypoglycemia unawareness is the leading modality for allogeneic islet transplantation [66, 67]. As we have discussed above, AHCLs are mitigating the risks of severe hypoglycemia, and the indications for ITA are likely to drop. Some patients will still be reluctant to be on a pump or will not respond to technology adequately, and will therefore remain *bonafide* candidates for an ITA. The other impact of AHCLs is not to prevent, but to slow down the progression of diabetic nephropathy, and thus increase the age at which patients with T1DM who develop chronic kidney failure will have to face renal replacement therapy. We will have to care for an increased population of older, frailer patients, who would have been ideal candidates for simultaneous pancreas-kidney transplantation at a younger age, with a better general condition and fewer cardio-vascular issues. These patients, if they are fit to receive a kidney transplant, and most of them will, should therefore be offered simultaneous islet-kidney (SIK) transplantation rather than remain on insulin, while being immunosuppressed anyway [68]. This will amount to an ironical “return to the future,” since SIK was by far the main modality of islet transplantation before the “Edmonton protocol” induced a paradigm shift in the world of beta cell replacement [69].

To summarize (Table 1), we foresee that, although they hold serious promise, regenerative medicine solutions still have a long way to go before being available to more than a lucky few patients with T1DM. Xenotransplantation of islets is a serious outsider that will face the same as yet unresolved issues as stem cells. We therefore believe that, in times where technology has measurably impacted the management of T1DM patients, but not to the point of offering a physiologic metabolic control, allogeneic islet transplantation still has several years of existence ahead. Indications for islet transplantation will undergo modifications, rather than see a decrease in activity. It is very likely that we will observe a diminution of the number of ITAs performed, but an increase in SIK activity, without a drop in overall islet transplant activity, and that “foretold death” of allogeneic islet transplantation will only be witnessed by the next-generation of diabetologists and transplant physicians.

**TABLE 1:** Comparison of management technologies for T1DM.

	Allogeneic islets	Stem-cells	Xenogeneic islets	Closed loops
Status	Standard-of-care	Phase III	Phase I/II	Standard-of-care
Glycemic control	Good	Good	Uncertain	Acceptable
Availability	Limited	Theoretically infinite	Theoretically infinite	Unlimited
Limiting factor	Organ donors	Bioreactor capacity	Breeding capacity	n.a.
Costs	High	Very high	High	Acceptable
Safety risks	Donor-derived infection or malignancy	Tumorigenicity: off-target growth, teratoma	Zoonosis	none
Immunology	Allorejection	Allorejection (ESC) Immunogenicity of autologous iPSC	Xenorejection	n.a.
Immune modulation	Encapsulation strategies Gene-editing	Encapsulation strategies Gene-editing	Encapsulation strategies Gene-editing	n.a.

ESC, embryonic stem cells; iPSC, induced pluripotent stem cells; n.a., not applicable.

## Data Availability

The original contributions presented in the study are included in the article/supplementary material, further inquiries can be directed to the corresponding author.

## Abbreviations

AHCL, advanced hybrid closed loop; ATMP, Advanced Medicinal Therapy Products; CGM, continuous glucose monitoring; ESC, embryonic stem cell; iPSC, induced pluripotent stem cell; ITA, islet transplant alone; PCMV/PRV, porcine CMV/porcine roseolovirus; PERV, porcine endogenous retrovirus; SIK, simultaneous islet-kidney transplantatio; T1DM, type 1 diabetes mellitus; TIR, time I range.

## References

[1] D'Amour KA Bang AG Eliazer S Kelly OG Agulnick AD Smart NG Production of Pancreatic Hormone-Expressing Endocrine Cells from Human Embryonic Stem Cells. Nat Biotechnol (2006) 24:1392–401. 10.1038/nbt1259 17053790

[2] Rezania A Bruin JE Arora P Rubin A Batushansky I Asadi A Reversal of Diabetes with Insulin-Producing Cells Derived In Vitro from Human Pluripotent Stem Cells. Nat Biotechnol (2014) 32:1121–33. 10.1038/nbt.3033 25211370

[3] Pagliuca FW Millman JR Gürtler M Segel M Van Dervort A Ryu JH Generation of Functional Human Pancreatic β Cells In Vitro. Cell (2014) 159:428–39. 10.1016/j.cell.2014.09.040 25303535 PMC4617632

[4] Russ HA Parent AV Ringler JJ Hennings TG Nair GG Shveygert M Controlled Induction of Human Pancreatic Progenitors Produces Functional Beta-like Cells In Vitro. EMBO J (2015) 34:1759–72. 10.15252/embj.201591058 25908839 PMC4516429

[5] Pellegrini S Chimienti R Scotti GM Giannese F Lazarevic D Manenti F Transcriptional Dynamics of Induced Pluripotent Stem Cell Differentiation into β Cells Reveals Full Endodermal Commitment and Homology with Human Islets. Cytotherapy (2021) 23:311–9. 10.1016/j.jcyt.2020.10.004 33246884

[6] Balboa D Barsby T Lithovius V Saarimäki-Vire J Omar-Hmeadi M Dyachok O Functional, Metabolic and Transcriptional Maturation of Human Pancreatic Islets Derived from Stem Cells. Nat Biotechnol (2022) 40:1042–55. 10.1038/s41587-022-01219-z 35241836 PMC9287162

[7] Du Y Liang Z Wang S Sun D Wang X Liew SY Human Pluripotent Stem-Cell-Derived Islets Ameliorate Diabetes in Non-human Primates. Nat Med (2022) 28:272–82. 10.1038/s41591-021-01645-7 35115708

[8] Henry RR Pettus J Wilensky J Shapiro AMJ Senior PS Roep B Initial Clinical Evaluation of VC-01TM Combination Product-A Stem Cell–Derived Islet Replacement for Type 1 Diabetes (T1D). Diabetes (2018) 67(Suppl. l_1):138–OR. 10.2337/db18-138-or

[9] Shapiro AMJ Thompson D Donner TW Bellin MD Hsueh W Pettus J Insulin Expression and C-Peptide in Type 1 Diabetes Subjects Implanted with Stem Cell-Derived Pancreatic Endoderm Cells in an Encapsulation Device. Cell Rep Med (2021) 2:100466. 10.1016/j.xcrm.2021.100466 35028608 PMC8714853

[10] Keymeulen B De Groot K Jacobs-Tulleneers-Thevissen D Thompson DM Bellin MD Kroon EJ Encapsulated Stem Cell-Derived β Cells Exert Glucose Control in Patients With Type 1 Diabetes. Nat Biotechnol (2024) 42:1507–14. 10.1038/s41587-023-02055-5 38012450 PMC11471599

[11] Vertex Press Release. Vertex Announces Positive Results From Ongoing Phase 1/2 Study of VX-880 for the Treatment of Type 1 Diabetes Presented at the American Diabetes Association 84th Scientific Sessions. (2024). Available online at: https://news.vrtx.com/news-releases/news-release-details/vertex-announces-positive-results-ongoing-phase-12-study-vx-880. (Accessed March 3, 2025).

[12] Vertex Press Release. Vertex Reports Third Quarter 2024 Financial Results (2024). Available online at: https://news.vrtx.com/news-releases/news-release-details/vertex-reports-third-quarter-2024-financial-results. (Accessed March 11, 2025).

[13] Wang S Du Y Zhang B Meng G Liu Z Liew SY Transplantation of Chemically Induced Pluripotent Stem-Cell-Derived Islets under Abdominal Anterior Rectus Sheath in a Type 1 Diabetes Patient. Cell (2024) 187:6152–64.e18. 10.1016/j.cell.2024.09.004 39326417

[14] Wu J Li T Guo M Ji J Meng X Fu T Treating a Type 2 Diabetic Patient with Impaired Pancreatic Islet Function by Personalized Endoderm Stem Cell-Derived Islet Tissue. Cell Discov (2024) 10:45. 10.1038/s41421-024-00662-3 38684699 PMC11058776

[15] Scholz H Sordi V Piemonti L. Cautious Optimism Warranted for Stem Cell-Derived Islet Transplantation in Type 2 Diabetes. Transpl Int (2024) 37:13358. 10.3389/ti.2024.13358 39131791 PMC11310020

[16] Zhao T Zhang ZN Rong Z Xu Y. Immunogenicity of Induced Pluripotent Stem Cells. Nature (2011) 474:212–5. 10.1038/nature10135 21572395

[17] Piemonti L. The Last Mile in Beta-Cell Replacement Therapy for Type 1 Diabetes: Time to Grow Up. Transpl Int 2025; 37:14565 10.3389/ti.2025.14565 PMC11998595 40236754

[18] Berishvili E Peloso A Tomei AA Pepper AR. The Future of Beta Cells Replacement in the Era of Regenerative Medicine and Organ Bioengineering. Transpl Int (2024) 37:12885. 10.3389/ti.2024.12885 38544564 PMC10966588

[19] Lei J Coronel MM Yolcu ES Deng H Grimany-Nuno O Hunckler MD FasL Microgels Induce Immune Acceptance of Islet Allografts in Nonhuman Primates. Sci Adv (2022) 8:eabm9881. 10.1126/sciadv.abm9881 35559682 PMC9106299

[20] Han X Wang M Duan S Franco PJ Kenty JH Hedrick P Generation of Hypoimmunogenic Human Pluripotent Stem Cells. Proc Natl Acad Sci U S A (2019) 116:10441–6. 10.1073/pnas.1902566116 31040209 PMC6535035

[21] Hu X White K Young C Olroyd AG Kievit P Connolly AJ Hypoimmune Islets Achieve Insulin Independence after Allogeneic Transplantation in a Fully Immunocompetent Non-human Primate. Cell Stem Cell (2024) 31:334–40.e5. 10.1016/j.stem.2024.02.001 38335966

[22] Sana Therapeutics Press Release (2025). Available online at: https://ir.sana.com/news-releases/news-release-details/sana-biotechnology-announces-positive-clinical-results-type-1. (Accessed March 12, 2025).

[23] Piemonti L Scholz H de Jongh D Kerr-Conte J van Apeldoorn A Shaw JAM The Relevance of Advanced Therapy Medicinal Products in the Field of Transplantation and the Need for Academic Research Access: Overcoming Bottlenecks and Claiming a New Time. Transpl Int (2023) 36:11633. 10.3389/ti.2023.11633 37822447 PMC10563816

[24] Berishvili E Piemonti L de Koning EJP Lindstedt S Scholz H Scott WE ESOT Roadmap for Advanced Therapy Medicinal Products in Transplantation: Navigating Regulatory Challenges to Enhance Access and Care. Transpl Int (2024) 37:13485. 10.3389/ti.2024.13485 39469665 PMC11513584

[25] Moon SJ Jung I Park CY. Current Advances of Artificial Pancreas Systems: A Comprehensive Review of the Clinical Evidence. Diabetes Metab J (2021) 45:813–39. 10.4093/dmj.2021.0177 34847641 PMC8640161

[26] Breton MD Kovatchev BP. One Year Real-World Use of the Control-IQ Advanced Hybrid Closed-Loop Technology. Diabetes Technol Ther (2021) 23(9):601–8. 10.1089/dia.2021.0097 33784196 PMC8501470

[27] Benhamou PY Adenis A Lebbad H Tourki Y Heredia MB Gehr B One-year Real-World Performance of the DBLG1 Closed-Loop System: Data from 3706 Adult Users with Type 1 Diabetes in Germany. Diabetes Obes Metab (2023) 25:1607–13. 10.1111/dom.15008 36751978

[28] Lablanche S Delagenière J Jalbert M Sonnet E Benichou M Arnold N 12-Month Real-Life Efficacy of the MiniMed 780G Advanced Closed-Loop System in Patients Living with Type 1 Diabetes: A French Observational, Retrospective, Multicentric Study. Diabetes Technol Ther (2024) 26:426–32. 10.1089/dia.2023.0414 38236643

[29] Rizzi A Tartaglione L Lucaccini Paoli L Leo ML Popolla V Viti L Evaluation of Time in Tight Range and the Glycaemia Risk Index in Adults With Type 1 Diabetes Using an Advanced Hybrid Closed Loop System: A 1-Year Real-World Assessment. Diabetes Obes Metab (2024) 26:4078–86. 10.1111/dom.15766 39010292

[30] Nathan DM. Realising the Long-Term Promise of Insulin Therapy: The DCCT/EDIC Study. Diabetologia (2024) 64:1049–58. 10.1007/s00125-021-05397-4 33550441

[31] Toppe C Möllsten A Waernbaum I Schön S Gudbjörnsdottir S Landin-Olsson M Decreasing Cumulative Incidence of End-Stage Renal Disease in Young Patients With Type 1 Diabetes in Sweden: A 38-Year Prospective Nationwide Study. Diabetes Care (2019) 42:27–31. 10.2337/dc18-1276 30352897

[32] Halliday JA Russell-Green S Lam B Trawley S McAuley SA Bach LA Six Months of Hybrid Closed-Loop Therapy Improves Diabetes-specific Positive Well-Being, and Reduces Diabetes Distress and Fear of Hypoglycemia: Secondary Analysis of a Randomized Controlled Trial. BMJ Open Diabetes Res Care (2024) 12:e004428. 10.1136/bmjdrc-2024-004428 PMC11683942 39797667

[33] Lal RA Basina M Maahs DM Hood K Buckingham B Wilson DM. One Year Clinical Experience of the First Commercial Hybrid Closed-Loop System. Diabetes Care (2019) 42(12):2190–6. 10.2337/dc19-0855 31548247 PMC6868462

[34] The Nobel Prize Website (2025). Available online at: https://www.nobelprize.org/prizes/medicine/1923/banting/lecture/. (Accessed March 5, 2025).

[35] Piemonti L. Felix Dies Natalis, Insulin Ceterum Autem Censeo Beta Is Better. Acta Diabetol (2021) 58(10):1287–306. 10.1007/s00592-021-01737-3 34027619

[36] Anderson DJ Jones-Carr M Perry J Kumar V Porrett PM Locke JE. Genetically Modified Porcine Kidneys Have Sufficient Tissue Integrity for Use in Pig-To-Human Xenotransplantation. Ann Surg (2024) 280:374–82. 10.1097/SLA.0000000000006380 38842179

[37] Moazami N Stern JM Khalil K Kim JI Narula N Mangiola M Pig-to-human Heart Xenotransplantation in Two Recently Deceased Human Recipients. Nat Med (2023) 29:1989–97. 10.1038/s41591-023-02471-9 37488288

[38] Schmauch E Piening B Mohebnasab M Xia B Zhu C Stern J Integrative Multi-Omics Profiling in Human Decedents Receiving Pig Heart Xenografts. Nat Med (2024) 30:1448–60. 10.1038/s41591-024-02972-1 38760586

[39] Griffith BP Grazioli A Singh AK Tully A Galindo J Saharia KK Transplantation of a Genetically Modified Porcine Heart into a Live Human. Nat Med (2025) 31:589–98. 10.1038/s41591-024-03429-1 39779924

[40] Kawai T Williams WW Elias N Fishman JA Crisalli K Longchamp A Xenotransplantation of a Porcine Kidney for End-Stage Kidney Disease. N Engl J Med (2025). 10.1056/NEJMoa2412747 39927618

[41] Berney T Naesens M Schneeberger S. Xenotransplantion: Defeating the “Shumway Curse” an Interview with Drs. Bartley Griffith, Jayme Locke, Robert Montgomery, and Bruno Reichart. Transpl Int (2022) 35:10439. 10.3389/ti.2022.10439 35431639 PMC9005640

[42] Ali A Kurome M Kessler B Kemter E Wolf E. What Genetic Modifications of Source Pigs Are Essential and Sufficient for Cell, Tissue, and Organ Xenotransplantation? Transpl Int (2024) 37:13681. 10.3389/ti.2024.13681 39697899 PMC11652200

[43] Markmann JF Bartlett ST Johnson P Korsgren O Hering BJ Scharp D Executive Summary of IPITA-TTS Opinion Leaders Report on the Future of β-Cell Replacement. Transplantation (2016) 100:e25–31. 10.1097/TP.0000000000001054 27082827

[44] Kim JM Park CG. Current Status of Pancreatic Islet Xenotransplantation. Clin Transpl Res (2025). 10.4285/ctr.24.0046 PMC11959427 39924969

[45] Fonseca LM Krause N Lebreton F Berishvili E. Recreating the Endocrine Niche: Advances in Bioengineering the Pancreas. Artif Organs (2025). 10.1111/aor.14950 39844747

[46] Chen CC Pouliquen E Broisat A Andreata F Racapé M Bruneval P Endothelial Chimerism and Vascular Sequestration Protect Pancreatic Islet Grafts from Antibody-Mediated Rejection. J Clin Invest (2018) 128:219–32. 10.1172/JCI93542 29202467 PMC5749508

[47] Chaigne B Geneugelijk K Bédat B Ahmed MA Hönger G De Seigneux S Immunogenicity of Anti-HLA Antibodies in Pancreas and Islet Transplantation. Cell Transpl (2016) 25:2041–50. 10.3727/096368916X691673 27196533

[48] Pouliquen E Baltzinger P Lemle A Chen CC Parissiadis A Borot S Anti-Donor HLA Antibody Response after Pancreatic Islet Grafting: Characteristics, Risk Factors, and Impact on Graft Function. Am J Transpl (2017) 17:462–73. 10.1111/ajt.13936 27343461

[49] Piemonti L Citro A Tomajer V Partelli S Caldara R. Pig Xenotransplantation in Beta Cell Replacement: Addressing Challenges and Harnessing Potential for Type 1 Diabetes Therapy. Transpl Int (2024) 37:13122. 10.3389/ti.2024.13122 39512630 PMC11540633

[50] Kim BJ Shin JS Min BH Kim JM Park CG Kang HJ Clinical Trial Protocol for Porcine Islet Xenotransplantation in South Korea. Diabetes Metab J (2024) 48:1160–8. 10.4093/dmj.2023.0260 38772544 PMC11621658

[51] Hawthorne WJ Salvaris EJ Chew YV Burns H Hawkes J Barlow H Xenotransplantation of Genetically Modified Neonatal Pig Islets Cures Diabetes in Baboons. Front Immunol (2022) 13:898948. 10.3389/fimmu.2022.898948 35784286 PMC9243461

[52] Sanatkar SA Kinoshita K Maenaka A Hara H Cooper DKC. The Evolution of Immunosuppressive Therapy in Pig-To-Nonhuman Primate Organ Transplantation. Transpl Int (2025) 37:13942. 10.3389/ti.2024.13942 39872238 PMC11770881

[53] Mourad NI Gianello P. Enhanced Insulin Production from Porcine Islets: More Insulin, Less Islets. Transpl Int (2024) 37:13954. 10.3389/ti.2024.13954 39744044 PMC11688178

[54] Tönjes RR. Aspects of Regulation of Xenotransplantation in Europe. Transpl Int (2024) 37:13349. 10.3389/ti.2024.13349 39726676 PMC11671226

[55] Denner J. Monitoring for PERV Following Xenotransplantation. Transpl Int (2024) 37:13491. 10.3389/ti.2024.13491 39434857 PMC11491343

[56] Längin M Bender M Schmoeckel M Reichart B. Progress in Orthotopic Pig Heart Transplantation in Nonhuman Primates. Transpl Int (2024) 37:13607. 10.3389/ti.2024.13607 39399753 PMC11466817

[57] Denner J. Role of a Porcine Herpesvirus, PCMV/PRV, in Xenotransplantation. Transpl Int (2025) 38:14087. 10.3389/ti.2025.14087 39967601 PMC11832308

[58] Mohiuddin MM Singh AK Scobie L Goerlich CE Grazioli A Saharia K Graft Dysfunction in Compassionate Use of Genetically Engineered Pig-To-Human Cardiac Xenotransplantation: A Case Report. Lancet (2003) 402:397–410. 10.1016/S0140-6736(23)00775-4 PMC10552929 37393920

[59] Tol MC de Bont DFA Boon WPC de Koning EJP van Apeldoorn AA. Preferred Islet Delivery Device Characteristics and Implantation Strategies of Patients with Type 1 Diabetes. Transpl Int (2023) 36:11077. 10.3389/ti.2023.11077 37908676 PMC10614671

[60] Lebreton F Lavallard V Bellofatto K Bonnet R Wassmer CH Perez L Insulin-producing Organoids Engineered from Islet and Amniotic Epithelial Cells to Treat Diabetes. Nat Commun (2019) 10:4491. 10.1038/s41467-019-12472-3 31582751 PMC6776618

[61] Wassmer CH Lebreton F Bellofatto K Bosco D Berney T Berishvili E. Generation of Insulin-Secreting Organoids: A Step toward Engineering and Transplanting the Bioartificial Pancreas. Transpl Int (2020) 33:1577–88. 10.1111/tri.13721 32852858 PMC7756715

[62] Honarpisheh M Lei Y Zhang Y Pehl M Kemter E Kraetzl M Formation of Re-aggregated Neonatal Porcine Islet Clusters Improves *In Vitro* Function and Transplantation Outcome. Transpl Int (2022) 35:10697. 10.3389/ti.2022.10697 36685665 PMC9846776

[63] Berney T Wassmer CH Lebreton F Bellofatto K Fonseca LM Bignard J From Islet of Langerhans Transplantation to the Bioartificial Pancreas. Presse Med (2022) 51:104139. 10.1016/j.lpm.2022.104139 36202182

[64] Vantyghem MC Raverdy V Balavoine AS Defrance F Caiazzo R Arnalsteen L Continuous Glucose Monitoring After Islet Transplantation in Type 1 Diabetes: An Excellent Graft Function (β-Score Greater Than 7) Is Required to Abrogate Hyperglycemia, whereas a Minimal Function Is Necessary to Suppress Severe Hypoglycemia (β-Score Greater Than 3). J Clin Endocrinol Metab (2012) 97:E2078–83. 10.1210/jc.2012-2115 22996144 PMC3485599

[65] Marfil-Garza BA Imes S Verhoeff K Hefler J Lam A Dajani K Pancreatic Islet Transplantation in Type 1 Diabetes: 20-year Experience from a Single-Centre Cohort in Canada. Lancet Diabetes Endocrinol (2022) 10:519–32. 10.1016/S2213-8587(22)00114-0 35588757

[66] Shapiro AM Lakey JR Ryan EA Korbutt GS Toth E Warnock GL Islet Transplantation in Seven Patients with Type 1 Diabetes Mellitus Using a Glucocorticoid-free Immunosuppressive Regimen. N Engl J Med (2000) 343:230–8. 10.1056/NEJM200007273430401 10911004

[67] Collaborative Islet Transplant Registry. Eleventh Allograft Report (2022). Available online at: https://www.citregistry.org/system/files/11th_CITR_Network_Report_Allograft_2022_0.pdf. (Accessed March 8, 2025).

[68] Wojtusciszyn A Branchereau J Esposito L Badet L Buron F Chetboun M Indications for islet or pancreatic transplantation: Statement of the TREPID working group on behalf of the Société francophone du diabète (SFD), Société francaise d'endocrinologie (SFE), Société francophone de transplantation (SFT) and Société française de néphrologie - dialyse - transplantation (SFNDT). Diabetes Metab (2019) 45:224–37. 10.1016/j.diabet.2018.07.006 30223084

[69] International Islet Transplant Registry. Newsletter #9 (2001). Available online at: https://www.med.uni-giessen.de/itr/newsletter/no_9/news_9.pdf. (Accessed March 8, 2025).

